# Developing a rapid predictive model for falls in older hospitalized patients

**DOI:** 10.3389/fpubh.2024.1421078

**Published:** 2024-10-02

**Authors:** Mengmeng Hu, Sujuan Lu, Jiangan Guan, Wenqian Deng, Yu Hu, Yao Huang, Keying Li, Mengdan He, Zhiyi Wang, Chan Chen, Xiufang Chen

**Affiliations:** ^1^Department of General Practice, The Second Affiliated Hospital and Yuying Children’s Hospital of Wenzhou Medical University, Wenzhou, China; ^2^Wenzhou Key Laboratory of Precision General Practice and Health Management, Wenzhou, China; ^3^Department of Geriatric Medicine, The First Affiliated Hospital of Wenzhou Medical University, Wenzhou, China; ^4^South ZheJiang Institute of Radiation Medicine and Nuclear Technology, Wenzhou Medical University, Wenzhou, China

**Keywords:** older patients, in-hospital falls, independent risk factors, predictive model, nomogram

## Abstract

**Background:**

This study was aimed to identify the independent risk factors for falls n hospitalized older patients and develop a corresponding predictive model.

**Methods:**

A retrospective observational study design was adopted, comprising 440 older patients with falls history and 510 older patients without falls history during hospitalization. Data collected included demographic information, vital signs, comorbidities, psychiatric disorder, function absent, current medication, other clinical indicators.

**Results:**

Mobility disability, high-risk medications use, frequency of hospitalizations, psychiatric disorder, visual impairment are independent risk factors for falls in older patients. The A-M2-HPV scoring system was developed. The AUC value of the nomogram was 0.884, indicating the model has excellent discriminative ability. The AUC value of the A-M2-HPV score was 0.788, demonstrating better discrimination and stratification capabilities.

**Conclusion:**

The A-M2-HPV scoring system provides a valuable tool to assess the risk of falls in hospitalized older patients and to aid in the implementation of preventive measures.

## Introduction

With the increase of older people population, the problem of falls among older people is becoming increasingly prominent, posing a global challenge (1). Approximately 1 million falls occur in hospitals each year, constituting about 70% of inpatient accidents and posing a significant threat to the health and safety of older people (2). Falls not only prolong the hospital stay of older people, increase the risk of complications, but also lead to a sharp rise in medical expenses (3). This situation not only burdens patients physically and mentally but also imposes significant economic pressure on the healthcare system. According to national disease surveillance data from 2015, falls have emerged as the primary cause of injury-related deaths among individuals aged 65 and over in China (4). A study involving more than 30,000 older people’s medical expenses data showed that the medical expenses attributed to falls were as high as about 50 billion US dollars (5). Therefore, early identification, prevention and management of falls are of great significance for reducing the burden of health care and improving patient’s quality of life.

Falls among older people can be prevented and controlled. Numerous studies have demonstrated that implementing fall prevention measures can reduce the risk of falls among hospitalized patients by up to 30% (6). Effective measures to prevent falls include using fall risk assessment scales to screen high-risk groups and providing early nursing interventions. However, the scales currently employed to assess the risk of falls among older people vary in predictive validity, and none has been universally recognized as the superior option (7). The St Thomas’s Risk Assessment Tool (STRATIFY) has stable and good predictive validity for assessing the risk of falls among older patients, but its internal consistency is low and its accuracy needs to be improved (8). The Hendrich II fall risk model (HFRM II) is concise and convenient, supported by many studies, but its multiple items may increase assessment time and the burden on healthcare workers (9). The Morse Fall Scale (MFS) is widely used and has good reliability and validity, but it mainly focuses on physiological factors and cannot predict falls caused by environmental, pharmacological, and psychological factors (10). The most important thing is that the above fall risk models need to be assessed after various examinations or tests, and cannot be evaluated for hospitalized patients immediately.

This study aims to establish a simple and efficient model to quickly identify older hospitalized patients at high risk for falls, and it only relies on some key indicators, which can be very convenient to obtain through consultation and physical examination when patients are admitted to the hospital.

## Methods

### Data sources

This study is a multicenter, retrospective study, retrospective, observational research, with data sourced from The Second Affiliated Hospital and Yuying Children’s Hospital of Wenzhou Medical University, The First Affiliated Hospital of Wenzhou Medical University, both of which are large tertiary hospitals located in Wenzhou, China. This study retrospectively collected data on older patients (age ≥65 years) admitted to the Second Affiliated Hospital and Yuying Children’s Hospital of Wenzhou Medical University who experienced falls during hospitalization from January 2017 to September 2022, totaling 440 patients. Additionally, a random selection of older patients admitted during the same period without a history of falls during hospitalization was also included, comprising a total of 510 patients. The data collected from the First Affiliated Hospital of Wenzhou Medical University will be used as external validation data for the period from January 2017 to December 2022.

### Variables

The study retrospectively collected the following data: (1) demographic information including age, sex; (2) vital signs including temperature, pulse, respiratory rate, systolic blood pressure and diastolic blood pressure at the first records after admission; (3) comorbidities including hypertension (HBP), diabetes (DM), chronic obstructive pulmonary disease (COPD), heart disease, stroke; (4) psychiatric disorder including delirium, irritability, dementia; (5) function absent including visual impairment, auditory impairment, mobility disability (gait instability or assistive devices aid in walking, such as walker, wheelchair and attendants); (6) current medication including high-risk medications (sedative-hypnotics, psychotropic medications, hypoglycemic drugs), antihypertensive drugs, diuretics, analgesics; (7) Other clinical indicators including indwelling catheter (gastric tube, urinary catheter, drainage tube, endotracheal intubation, central venous catheterization), frequency of hospitalizations within 5 years, history of fall within the prior 6 months, St Thomas’s Risk Assessment Tool (STRATIFY) and Morse Fall Scale (MFS).

### Statistical analysis

All variables were subjected to univariate logistic regression analysis, and those with a *p* < 0.2 were included in the multivariate logistic regression analysis to identify the independent risk factors for falls during hospitalization among older patients. Continuous variables within the risk factors were stratified into categorical variables based on the interquartile range and in accordance with clinical application, followed by subsequent multivariable logistic regression analysis. For older people falls model, backward stepwise logistic regression based on the minimum Akaike information criterion (AIC) value was chosen to identify independent risk variables for falls. In addition, the variance inflation factor (VIF) of each predictor variable was used to check for multicollinearity between variables, with a VIF ≥5 indicating the presence of multicollinearity between variables. The predictive model was constructed by generating risk scores based on the regression coefficients of each predictor variable. The model assigned an integer or half-integer score by designating the variable with the smallest regression coefficient as the baseline with a score of 1 and calculating the scores for all other predictor variables by dividing their respective regression coefficients by the smallest one.

Receiver operating characteristic (ROC) curve and calibration curves were used to evaluate the differentiation and calibration abilities of this model. The model was retested for internal validation using bootstrap, with 1,000 bootstrap replicates. Statistical descriptions and analyses in this study were conducted using R (version4.2.3), with a *p*-value of less than 0.05 deemed to indicate statistical significance.

## Results

### Populations

A total of 950 older patients meeting the criteria were included in this study, of which 440 cases experienced falls. The mean age of the fallers was higher, and most patients had similar underlying comorbidities, with the exception of diabetes mellitus among the fallers. Additionally, a greater proportion of fallers presented with psychiatric disorders, visual impairments, mobility disability, high-risk medications use and higher frequency of hospitalizations within the past 5 years. The scores for MSF and the STRATIFY were also higher for falls group, with further details provided in Table 1.

**Table 1:** Demographic and clinical characteristics.

Variables	Non-falls (*N* = 510)	Falls (*N* = 440)	*p*-value
Demographics and social history			
Age, *n* (%)			<0.001
≥65–<75	314 (61.6%)	157 (35.7%)	
≥75–<85	161 (31.6%)	161 (36.6%)	
≥85	35 (6.86%)	122 (27.7%)	
Male sex, *n* (%)	299 (58.6%)	254 (57.7%)	0.779
Vital signs			
Temperature, median (IQR)	36.7 [36.6; 37.0]	36.6 [36.1; 37.1]	0.001
Pulse, median (IQR)	78.5 [70.0; 82.0]	78.0 [74.0; 88.0]	0.059
Respiratory rate, median (IQR)	20.0 [20.0; 20.0]	19.0 [16.0; 21.0]	0.001
Systolic BP, median (IQR)	154 [136; 167]	150 [131; 164]	0.013
Diastolic BP, median (IQR)	79.0 [72.0; 89.0]	80.5 [70.8; 93.0]	0.064
Comorbidity			
Hypertension, *n* (%)	328 (64.3%)	274 (62.3%)	0.560
Diabetes, *n* (%)	126 (24.7%)	150 (34.1%)	0.002
COPD, *n* (%)	34 (6.67%)	39 (8.86%)	0.252
Heart disease, *n* (%)	123 (24.1%)	119 (27.0%)	0.338
Stroke, *n* (%)	121 (23.7%)	101 (23.0%)	0.839
Conscious state			
Psychiatric disorder, *n* (%)	34 (6.67%)	135 (30.7%)	<0.001
Delirium, *n* (%)	5 (0.98%)	46 (10.5%)	<0.001
Irritability, *n* (%)	14 (2.75%)	69 (15.7%)	<0.001
Dementia, *n* (%)	15 (2.94%)	90 (20.5%)	<0.001
Function absent			
Visual impairment, *n* (%)	184 (36.1%)	330 (75.0%)	<0.001
Auditory impairment, *n* (%)	301 (59.0%)	284 (64.5%)	0.093
Mobility disability, *n* (%)	223 (43.7%)	337 (76.6%)	<0.001
Medication use			
High-risk medications, *n* (%)	110 (21.6%)	230 (52.3%)	<0.001
Sedative-hypnotics, *n* (%)	20 (3.92%)	90 (20.5%)	<0.001
Psychotropic medications, *n* (%)	3 (0.59%)	82 (18.6%)	<0.001
Hypoglycemic drugs, *n* (%)	101 (19.8%)	142 (32.3%)	<0.001
Antihypertensive drugs, *n* (%)	276 (54.1%)	216 (49.1%)	0.139
Diuretics, *n* (%)	22 (4.31%)	72 (16.4%)	<0.001
Analgesics, *n* (%)	38 (7.45%)	37 (8.41%)	0.671
Frequency of hospitalizations within 5 years, *n* (%)			<0.001
0	156 (30.6%)	31 (7.05%)	
>1, ≤3	318 (62.4%)	255 (58.0%)	
≥4	36 (7.06%)	154 (35.0%)	
History of fall within the prior 6 months, *n* (%)	256 (50.2%)	234 (53.2%)	0.394
STRATIFY, median (IQR)	1.00 [1.00; 2.00]	3.00 [1.00; 3.00]	<0.001
MSF, median (IQR)	50.0 [35.0; 60.0]	55.0 [30.0; 70.0]	0.110

Continuous data are presented as median (interquartile range), whereas categorical data are presented as frequency (percentage).

### Predictors of falls

**Figure 1:**
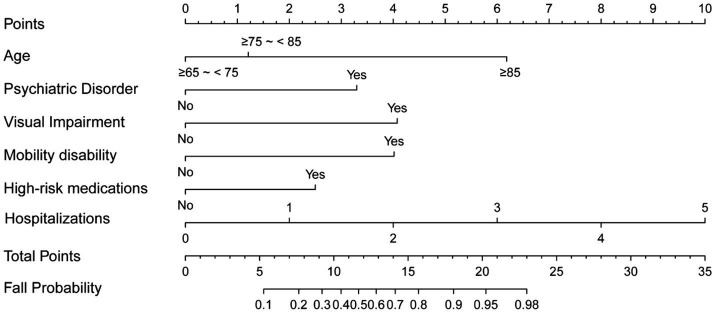
Nomogram to predicted fall risk in older patients.

Univariate regression analysis was conducted to test potential risk factors associated with falls. Those variables that exhibited significant predictive power in the univariate analysis were then selected for inclusion in multivariate regression analysis. The analysis identified psychiatric disorder, visual impairment, mobility disability, high-risk medications use, and frequency of hospitalizations was maintained consistently effects on the risk of falls (Supplementary Table S1; Table 2). Based on these fall risk indicators, a Nomogram was constructed (Figure 1).

**Table 2:** Prediction model of fall risk in older patients during hospitalization.

Variable	*β*	OR (95% CI)^a^	*p*-value	Point^b^
Age, y				
≥65–<75	—	—	—	—
≥75–<85	0.309	1.36 (0.93–2.00)	0.116	0
≥85	2.051	7.78 (4.53–13.36)	<0.001	2.5
Psychiatric disorder	1.170	3.22 (1.90–5.47)	<0.001	1.5
Visual impairment	1.386	4.00 (2.81–5.70)	<0.001	1.5
Mobility disability	1.351	3.86 (2.70–5.53)	<0.001	1.5
High-risk medications	0.832	2.30 (1.61–3.27)	<0.001	1
Frequency of hospitalizations within 5 years				
>1, ≤3	1.326	3.76 (2.27–6.24)	<0.001	1.5
≥4	3.129	22.85 (11.95–43.70)	<0.001	4
Total score				0 to 13.5

OR, odds ratio.

a Fall risk odds ratio.

b Assignment of points to risk factors was based on a linear transformation of the corresponding *β* regression coefficient. The coefficient of each variable was divided by 0.832 (the smallest absolute *β* value, high-risk medications) and allocated an integer or half integer score for each variable.

### A-M2-HPV score

To improve clinical utility, the continuous variable representing the frequency of hospitalizations was converted into categorical variables based on quartile distribution, and a corresponding scoring system was developed (age, mobility disability, high-risk medications use, frequency of hospitalizations, psychiatric disorder, visual impairment, A-M_2_-HPV). Individual patient scores were calculated by aggregating the points assigned to each identified prognostic factor, yielding a total score that varied from 0 to 13.5 (Table 2).

### Validation

**Figure 2:**
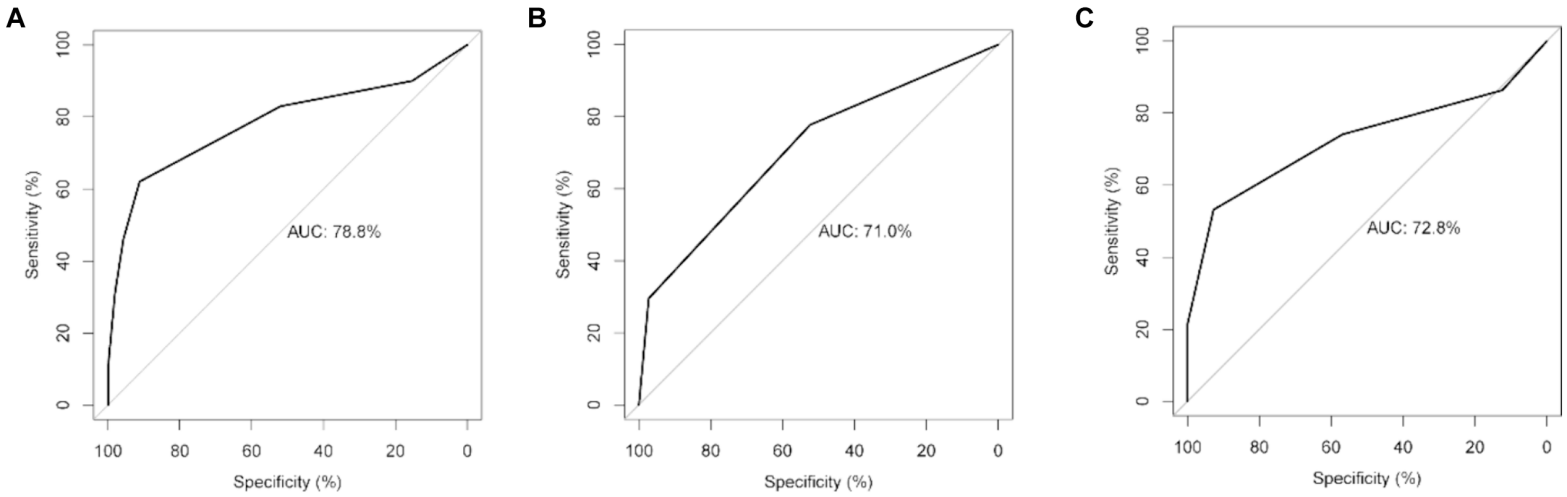
Receiver operating characteristic (ROC) of the A-M2-HPV score **(A)**, MSF score **(B)** and STRATIFY **(C)** predicted fall risk in older patients.

**Figure 3:**
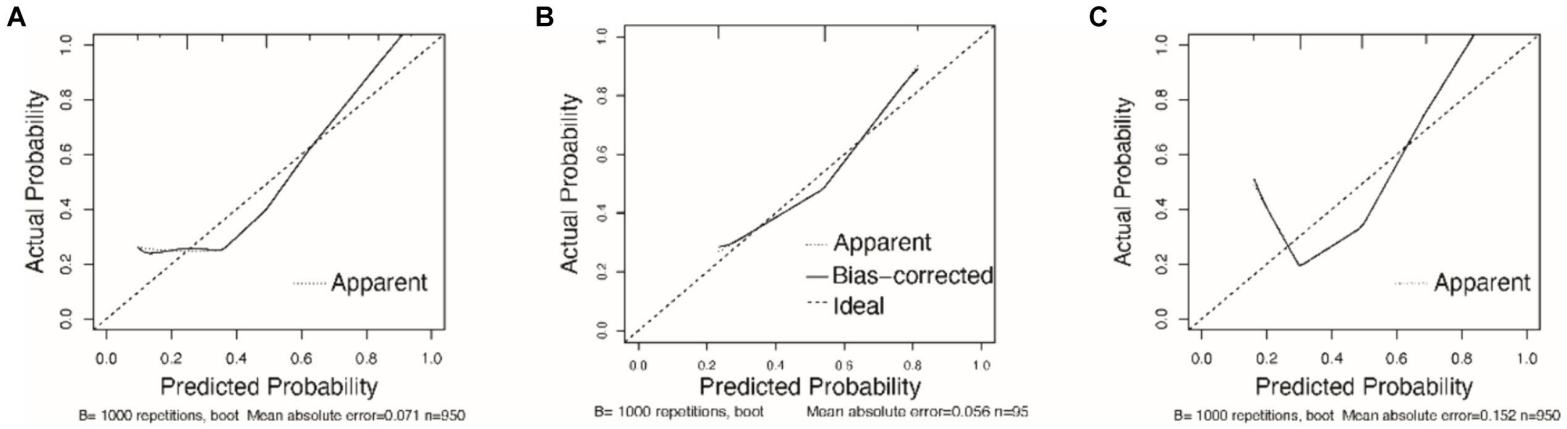
Calibration curves of the A-M2-HPV score **(A)**, MSF score **(B)** and STRATIFY **(C)** predicted fall risk in older patients.

**Figure 4:**
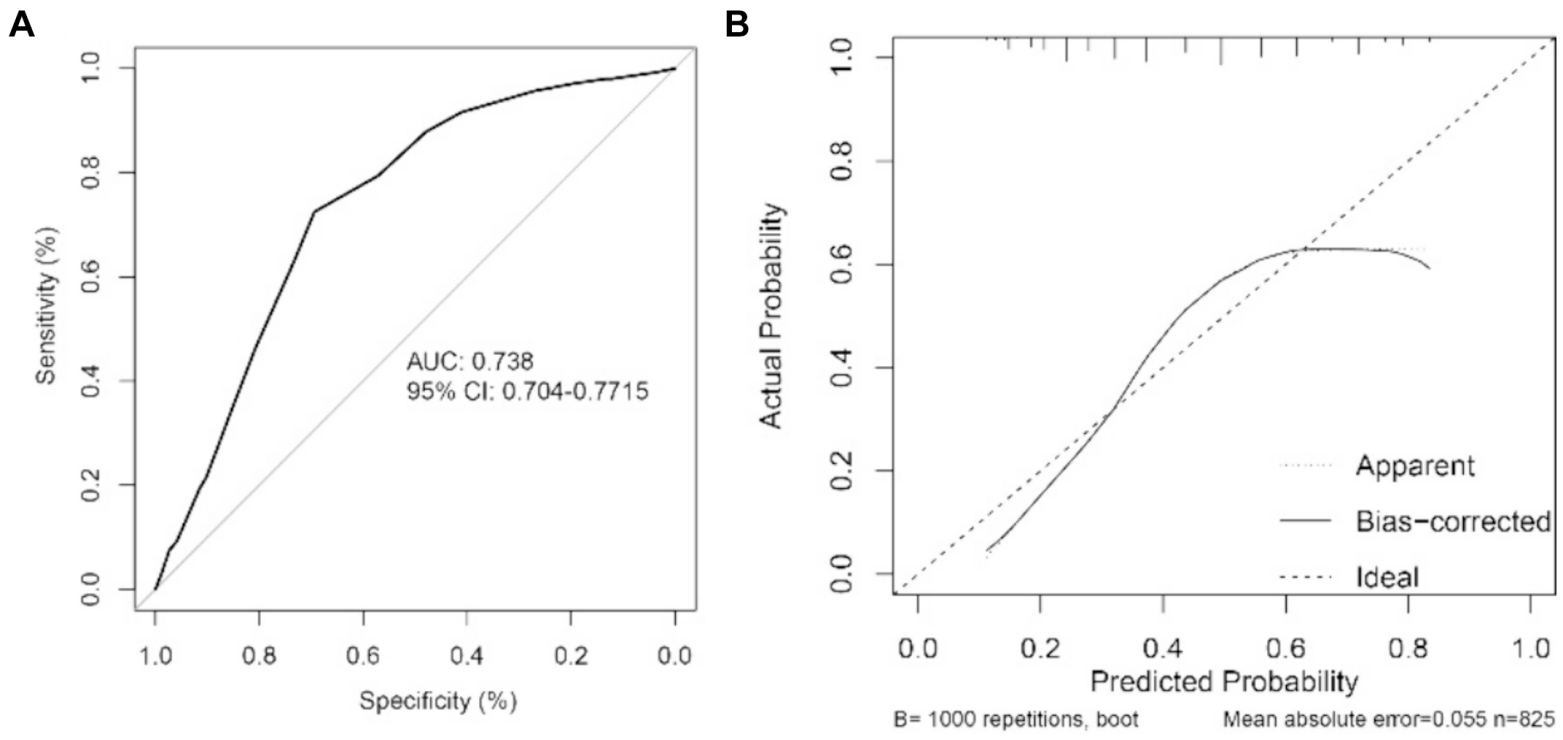
ROC curves **(A)** and calibration curves **(B)** of the A-M2-HPV score predicted fall risk in external validation.

The AUC value for the Nomogram was 0.884 (95% CI, 0.862–0.906), the calibration accuracy of the Nomogram was evaluated using calibration curves. The bias-corrected curve, derived from a bootstrap resampling approach, demonstrated a good agreement between the predicted probability and the actual probability (Supplementary Figure S1). We compared the performance of A-M_2_-HPV score with MSF, and STRATIFY for predicting fall probability in older patients. The AUC value for the A-M_2_-HPV score was 0.788 (95% CI, 0.757–0.820) which was significantly higher than that for MSF score 0.710 (95 CI, 0.680–0.740) and STRATIFY 0.728 (95% CI, 0.694–0.761) (Figure 2), indicating that the M_2_-HPV score had better discrimination than MSF and STRATIFY. Internal validation using the bootstrap method demonstrated that the AUC value was similar, with a value of 0.778 (95% CI, 0.749–0.808). Calibration plots revealed that the calibration accuracy of the A-M_2_-HPV score was significantly better than the MSF and STRATIFY (Figure 3). External validation showed that there was still good discrimination with an AUC value of 0.738 (95% CI, 0.704–0.772). The calibration plot showed that the calibration curve closely matched the ideal curve (Figure 4).

## Discussion

In this study, we constructed a predictive model for the risk of falls in older hospitalized patients based on simple clinical assessments. Psychiatric disorder was a risk factor in our research. Cognitive impairments caused by psychiatric disorders are a risk factor contributing to the high prevalence of fall incidents among older patients (11). Decreased mobility was another risk factor leading to falls in older hospitalized patients. Towne et al. (12) indicated that older patients requiring assistance with daily mobility were more prone to falls. Our study also found that visual impairment was closely related to the risk of falls in older hospitalized patients. Visual impairment can lead to a decline in older people patients’ balance and gait ability, thereby increasing their risk of falling. This is due to the fact that visual-motor signals provide direct information about head movements (13–15).

Moreover, the use of high-risk medications, including sedative-hypnotics, psychotropic medications, hypoglycemic drugs, etc., especially the use of sedative-hypnotics, increases the risk of falls in older hospitalized patients. Previous research has reported that older patients using sedative-hypnotics are at risk of injurious falls (16). This may be attributed to the impact of these medications on the central nervous system, which can easily lead to adverse reactions such as drowsiness, impaired balance, cognitive dysfunction, and motor deficits, especially in older patients (17, 18). It is worth noting that antihypertensive drugs are often considered related to falls in older patients, but our study did not confirm this. Therefore, the evidence that patients’ falls are directly caused by the use of antihypertensive drugs is unclear.

What sets us apart from other studies is that, in evaluating the overall health status of patients, we only use the number of hospitalizations in the past 5 years, instead of the various laboratory test indicators used in traditional assessment models to assess organ function. Compared to the complexity and diversity of the latter, the number of hospitalizations within a certain time frame can more simply and objectively reflect the patient’s physical health from a macro perspective, holding high predictive value. This indicator also constitutes the innovation of our study. Unlike other existing predictive model (16), our study includes only five simple clinical assessment indicators that can be obtained immediately upon admission without the need for laboratory tests. In fact, a high risk of falling does not necessarily mean that a fall has actually occurred. To some extent, falls are randomly incident within the short and variable duration of hospital stays, making early prevention of high-risk falls particularly important.

Compared to other traditional fall risk assessment scales for older people, such as the STRATIFY and MFS, our predictive scoring model does not have inferior sensitivity and specificity (19, 20). In fact, our predictive scoring model is based on only five simple clinical judgments and can effectively assess the fall risk of hospitalized older patients. Compared to traditional scales, it is easier to assess and no less practical. Unlike the MFS, the presence of an indwelling catheter was not considered a risk factor in our study. This may be because individuals with such catheters are more restricted and cautious when walking, due to the limitations imposed by the catheter, thereby reducing the risk of falls. In addition, unlike the Johns Hopkins Fall Risk Assessment Tool (JHFRAT) (21), age was not included in our predictive model. A possible explanation is that the factor of age is already included within the influence of hospitalization frequency. The older the individual, the more underlying diseases they have, and consequently, the higher their frequency of hospitalization. This is implicitly taken into account in the model without the need for explicit age categorization.

Nomogram and clinical scoring systems, because of their simplicity and ease of assessment, have been widely used in the field of medical research (22–24). In this study, we developed a clinical scoring predictive model and used the nomogram to intuitively present the risk of falls in older hospitalized patients. An important highlight of this study is the recognition of the randomness of fall incidents and the importance of early prevention. The indicators included in our predictive model can be assessed immediately upon admission. From the perspective of safety management, the purpose of using this predictive model is not only to forecast the occurrence of fall events but, more importantly, to implement effective preventive measures early on to reduce the risk of falls. Therefore, our predictive model includes simple clinical assessment indicators that can be evaluated right at the time of admission. This will help to more accurately assess the fall risk of older patients, enhance healthcare providers’ awareness of patient safety, and thus significantly and effectively reduce the incidence of falls among hospitalized older patients (25, 26).

From a public health perspective, the implications of our findings are significant. Falls among older patients not only lead to increased healthcare utilization and costs but also contribute to a decrease in the quality of life for older adults. By identifying individuals at high risk of falls, our predictive model can facilitate targeted interventions that can reduce the incidence of falls and associated injuries. This can lead to a significant reduction in the burden on healthcare systems, both in terms of direct medical costs and the indirect costs associated with long-term care and loss of productivity. Additionally, the early identification and management of fall risk can help in the development of policies and programs aimed at improving the safety and well-being of older adults in various settings, including hospitals, nursing homes, and community care facilities.

There are several limitations to our research. Firstly, the patients in this study were conducted in one center of China, and the sample size was not large, due to different geographical and ethnic diversities, this scoring model still needs further external validation. Second, the study data were collected retrospectively and did not assess the impact of certain patient variables, such as prevailing sleep conditions and anxiety states, as well as environmental, economic, and social support systems on falls. These factors may have had an effect on the occurrence of falls, but could not be confirmed.

## Conclusion

In summary, our study has developed a simple scoring model for predicting the risk of falls in hospitalized older patients. This model is not inferior to the MFS and STRATIFY, and it can be assessed immediately upon admission, which has guiding significance for clinical prevention of falls in older patients. The implementation of our predictive model in clinical practice can help to potentially reduce the burden of fall-related injuries and enhance the safety of care for older patients in hospital settings, thereby contributing to public health benefits.

## Data Availability

The original contributions presented in the study are included in the article/Supplementary material, further inquiries can be directed to the corresponding authors.
