# Supplementary material for: Developing a rapid predictive model for falls in older hospitalized patients
Source: Front Public Health. 2024 Oct 2;12:1421078. doi: 10.3389/fpubh.2024.1421078 (PMC11480073; doi:10.3389/fpubh.2024.1421078)
Supplement: Supplementary file 1 [file Data_Sheet_1.docx]

**Figure S1：Calibration curves of the nomogram predicted fall risk in older patients.**

**Supplemental Table 1: Univariate logistic regression**

| Variables | **OR (95%CI)** | ***P*** |
| --- | --- | --- |
| **Demographics and social history** |  |  |
| Age, Median (IQR, years) | 1.03 [1.01;1.05] | <0.001 |
| Male sex, n (%) | 0.96 [0.74;1.25] | 0.779 |
| **Vital signs** |  |  |
| Temperature, Median (IQR) | 0.68 [0.53;0.89] | 0.004 |
| Pulse, Median (IQR) | 1.02 [1.01;1.03] | 0.002 |
| Respiratory rate, Median (IQR) | 0.79 [0.74;0.84] | <0.001 |
| Systolic BP, Median (IQR) | 0.99 [0.99;1.00] | 0.045 |
| Diastolic BP, Median (IQR) | 1.00 [1.00;1.01] | 0.886 |
| **Comorbidity** |  |  |
| Hypertension, n (%) | 0.92 [0.70;1.19] | 0.516 |
| Diabetes, n (%) | 1.58 [1.19;2.09] | 0.002 |
| COPD, n (%) | 1.36 [0.84;2.21] | 0.209 |
| Heart disease, n (%) | 1.17 [0.87;1.56] | 0.303 |
| Stroke, n (%) | 0.96 [0.71;1.30] | 0.781 |
| **Conscious State** |  |  |
| Psychiatric disorder, n (%) | 6.17 [4.17;9.36] | 0.000 |
| Delirium, n (%) | 11.4 [4.94;33.7] | <0.001 |
| Irritability, n (%) | 6.52 [3.72;12.3] | <0.001 |
| Dementia, n (%) | 8.40 [4.92;15.4] | 0.000 |
| **Function absent, n (%)** |  |  |
| Visual impairment, n (%) | 5.30 [4.01;7.05] | 0.000 |
| Auditory impairment, n (%) | 1.26 [0.97;1.65] | 0.081 |
| Mobility disability, n (%) | 4.20 [3.18;5.59] | 0.000 |
| **Medication use** |  |  |
| High-risk medications, n (%) | 3.37 [2.56;4.44] | 0.000 |
| Sedative-hypnotics, n (%) | 6.25 [3.85;10.6] | <0.001 |
| Psychotropic medications, n (%) | 36.8 [13.6;156] | 0.000 |
| Hypoglycemic drugs, n (%) | 1.93 [1.44;2.60] | <0.001 |
| Antihypertensive drugs, n (%) | 0.82 [0.63;1.06] | 0.123 |
| Diuretics, n (%) | 4.31 [2.67;7.25] | <0.001 |
| Analgesics, n (%) | 1.14 [0.71;1.83] | 0.587 |
| Frequency of hospitalizations within 5 years, n (%) |  | 0.000 |
| 0 |  |  |
| >1, <=3 | 4.01 [2.67;6.21] |  |
| ≥4 | 21.2 [12.7;36.6] |  |
| History of fall within the prior 6 months, n (%) | 1.13 [0.87;1.46] | 0.360 |
| STRATIFY, Median (IQR) | 2.25 [1.97;2.57] | <0.001 |
| MSF, Median (IQR) | 1.01 [1.00;1.01] | 0.022 |
